# Augmenting SOFA-2 with immune markers to capture dysregulated host response in patients with suspected infection

**DOI:** 10.3389/fimmu.2026.1904045

**Published:** 2026-07-23

**Authors:** Yu Qi, Huihui Sun, Chunhong Hou

**Affiliations:** 1 Cheeloo College of Medicine, Shandong University, Jinan, China; 2 Heze Hospital Affiliated to Shandong First Medical University, Heze Municipal Hospital, Heze, China

**Keywords:** immune dysfunction, NLR (neutrophil-to-lymphocyte ratio), sepsis, sequential organ failure assessment, suspected infection

## Abstract

**Introduction:**

The updated SOFA-2 score excludes the immune system due to specificity concerns, creating a conceptual gap given that sepsis is defined by a dysregulated host response. We hypothesized that augmenting SOFA-2 with widely available immune markers—white blood cell counts, lymphocyte counts, and the neutrophil-to-lymphocyte ratio (NLR)—would improve mortality prediction in patients with suspected infection.

**Methods:**

We analyzed 75,203 adult patients from the MIMIC-IV, MIMIC-III, and eICU databases. We constructed an immune-augmented SOFA-2 model using provisional consensus criteria for cell counts and data-driven thresholds for NLR derived via generalized additive models. Discrimination was evaluated using random-effects meta-analysis of the area under the receiver operating characteristic (AUROC) curve and net reclassification improvement (NRI).

**Results:**

The immune-augmented model significantly outperformed standard SOFA-2 across all cohorts. In the pooled analysis, the immune-augmented SOFA-2 yielded a higher AUROC (0.750 [95% CI, 0.715–0.785]) compared to standard SOFA-2 (0.740 [95% CI, 0.712–0.768]; p=0.003). Additionally, incorporating immune markers improved clinical risk stratification, resulting in a significant pooled total NRI of 0.046 (95% CI, 0.014–0.078; p=0.005).

**Conclusions:**

Excluding the immune system limits the ability of the SOFA-2 score to capture the dysregulated host response essential to severe infection. Integrating immune markers significantly enhances prognostic accuracy and risk reclassification for patients with suspected infection.

## Introduction

The recently updated Sequential Organ Failure Assessment (SOFA-2) score aims to reflect contemporary critical care practices that focus on the severity of organ dysfunction. However, the immune system is excluded from SOFA-2 due to perceived limitations in biomarker specificity (1, 2). Given that sepsis is defined as a dysregulated host response that includes immune dysfunction (3, 4), this exclusion represents a conceptual gap. Our previous work demonstrated that the loss of lymphocytes and immune imbalance as critical features of sepsis progression distinct from physiological stress alone (5, 6). Thus, we hypothesized that incorporating widely available immune markers—white blood cell (WBC) counts, lymphocyte counts, and the neutrophil-to-lymphocyte ratio (NLR)—along with SOFA-2 might improve mortality prediction and risk classification in patients with suspected infection.

## Methods

### Data sources and study population

This multicenter retrospective cohort study utilized data from three critical care databases: MIMIC-IV (v3.1) (7), the CareVue subset of MIMIC-III (v1.4) (8), and the eICU Collaborative Research Database (v2.0) (9). We included adult patients (age ≥ 18 years) with suspected infection identified within 24 hours before or after intensive care unit (ICU) admission. For patients with multiple ICU admissions, only the first admission was analyzed. Institutional review board approval was waived due to the use of published deidentified data.

The operational definition of suspected infection was adapted based on data availability. In the MIMIC-IV and MIMIC-III cohorts, suspected infection was defined as the co-occurrence of body fluid cultures and antibiotic administration. Specifically, consistent with Sepsis-3 operational criteria, antibiotics were required to be administered within 24 hours prior to the culture timestamp or within 72 hours thereafter (10). In the eICU cohort, due to the unavailability of microbiological culture records, suspected infection was defined as the administration of two or more antibiotics doses within a 24-hour period. The full list of antibiotics used to define suspected infection is provided in **Supplementary Table 1**.

### Outcomes

The primary outcome was in-hospital mortality; the secondary outcome was 30-day mortality, assessed in the MIMIC-III and MIMIC-IV cohorts only, given the absence of post-discharge follow-up data in the eICU database.

### Variable definitions and score construction

The standard SOFA-2 score was calculated using the worst physiological and laboratory values recorded during the first 24 hours of ICU admission, adhering to the methodology described by Ranzani et al. (1).

To construct the immune-augmented SOFA-2 models, we incorporated three immune markers: WBC counts, lymphocyte counts, and the NLR. Scoring thresholds for WBC and lymphocyte counts were adopted directly from the provisional immune criteria proposed by the SOFA-2 Consensus Task Force (1). For NLR, given the lack of established consensus thresholds for organ failure scoring, we derived data-driven cutoffs using the MIMIC-IV cohort, which was randomly partitioned 7:3 into a training set and a test set. Within the training set, we fitted a generalized additive model (GAM) with penalized smoothing splines to characterize the non-linear relationship between NLR and the log-odds of in-hospital mortality. Based on the resulting risk curve, NLR values were stratified into 5 ordinal categories (0 to 4 points) corresponding to increasing mortality risk, with the derived cutoff values summarized in **Supplementary Table 2**. Model performance for MIMIC-IV was subsequently evaluated in the held-out test set to avoid optimism bias from using the same data for both cutoff derivation and evaluation. Missing proportions for WBC counts, lymphocyte counts, and NLR across all cohorts are summarized in **Supplementary Table 3**. Consistent with the original SOFA-2 derivation framework (2), missing values for WBC count, lymphocyte count, or NLR were assigned 0 points rather than imputed or used as a basis for case exclusion. The conventional SOFA-2 score retained its original range of 0 to 24 points. Each immune augmentation strategy was implemented as a single additional immune domain scored from 0 to 4 points according to the predefined criteria shown in **Supplementary Table 3**, resulting in a total possible score of 0 to 28 points for each augmented SOFA-2 model.

### Statistical analysis

Baseline characteristics were compared across cohorts, with continuous variables reported as median [IQR] and categorical variables as frequency (%). To account for the differing score ranges (0–24 for standard SOFA-2 vs 0–28 for immune-augmented models), we fitted logistic regression models for in-hospital mortality using standardized SOFA scores (Z-score transformation) to facilitate the comparison of odds ratios (OR) per standard deviation increase. Discrimination was evaluated using the area under the receiver operating characteristic curve (AUROC), and differences in AUROCs were tested using DeLong’s test.

The clinical utility of adding immune markers was assessed using the net reclassification improvement (NRI). We calculated categorical NRI using *a priori* risk thresholds of 10% and 40%. These cutoffs were selected to align with the mortality strata implied by the Sepsis-3 definitions, corresponding to a mortality risk of approximately 10% for sepsis (SOFA ≥ 2) and greater than 40% for septic shock (3).

To synthesize evidence across the three heterogeneous databases, we performed a two-stage meta-analysis. Estimates were first calculated within each cohort and then pooled using a random-effects model to account for between-study heterogeneity. All analyses were performed using R version 4.4.1.

## Results

The combined cohort included 72,839 patients (eICU: n=23,250; MIMIC-III: n=6143; MIMIC-IV: n=43,446). Baseline characteristics exhibited significant heterogeneity across cohorts. In-hospital mortality rates were 13%, 22%, and 12%, respectively, for the three datasets (**Supplementary Table S4**). The distributions of SOFA-1 and SOFA-2 scores in each cohort are shown in **Supplementary Figure 1**.

**Figure 1:**
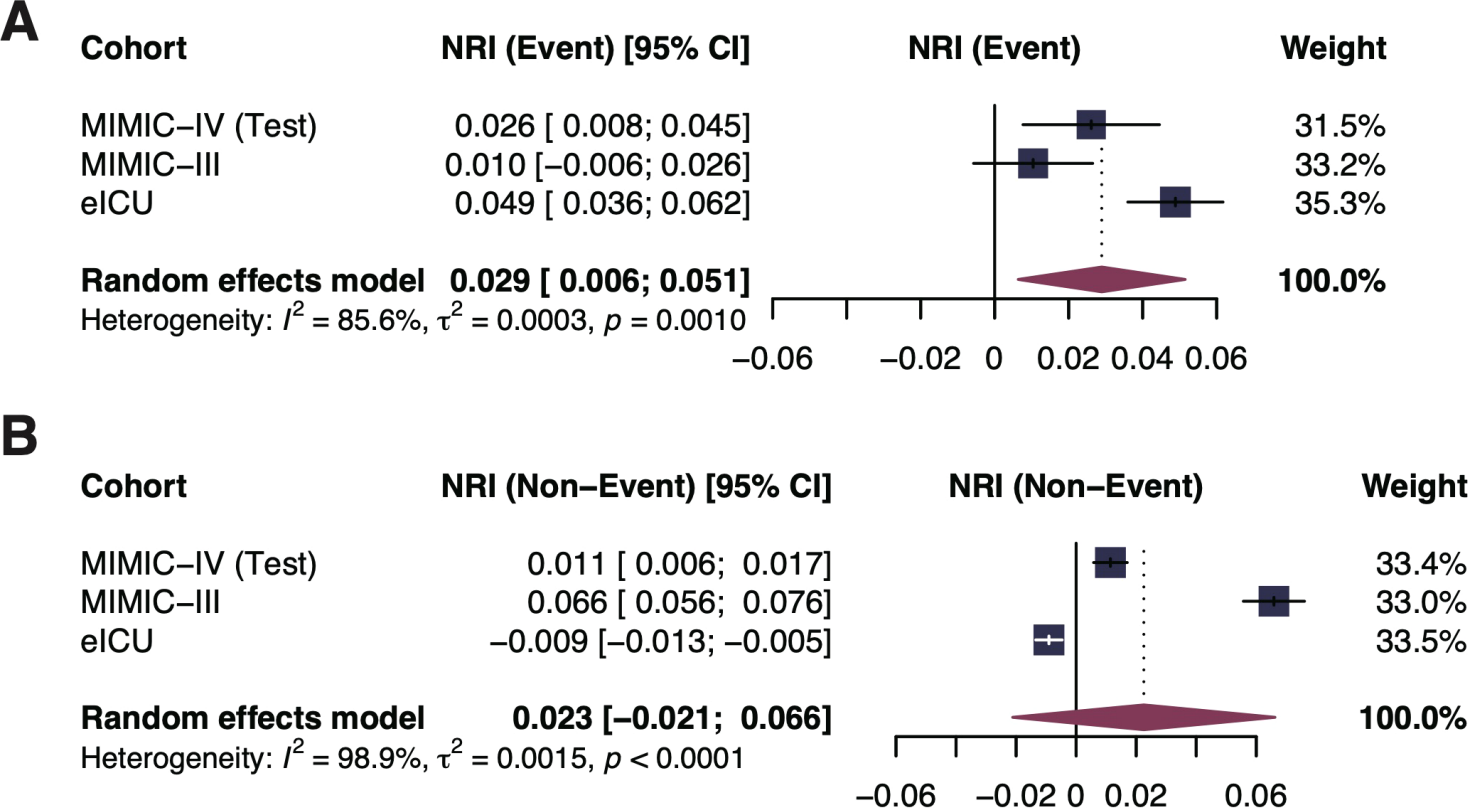
Net reclassification improvement of immune-augmented SOFA-2 versus SOFA-2 across 3 cohorts. **(A)** Forest plot of net reclassification improvement (NRI) for events (hospital nonsurvivors). **(B)** Forest plot of NRI for non-events (hospital survivors). The comparison evaluated the addition of immune markers to the standard SOFA-2 score in the MIMIC-IV, MIMIC-III, and eICU databases. Risk categories were defined as low (<10%), medium (10%-40%), and high (>40%) probability of in-hospital mortality. Diamonds represented the pooled estimates derived from random-effects models; squares indicated point estimates for each cohort with 95% CIs.

The standard SOFA-2 score showed variable performance or predicting in-hospital mortality compared with SOFA-1. Notably, SOFA-1 yielded a statistically higher AUC than SOFA-2 in both MIMIC-IV (0.768 [95% CI, 0.756 -0.781] vs 0.747 [95% CI, 0.734 -0.760]) and MIMIC-III (0.724 [95% CI, 0.709 -0.740] vs 0.714 [95% CI, 0.698 -0.730]), while SOFA-2 performed better in eICU (0.757 [95% CI, 0.748 -0.767] vs 0.763 [95% CI, 0. 754-0.772]) (**Table 1**). Given the omission of immune dysfunction from SOFA-2, we next evaluated immune-augmented versions of the score. **Supplementary Figure 2** summarizes the observed mortality-risk gradients across WBC count and NLR. Across cohorts, the immune-augmented models consistently improved predictive performance. In the pooled meta-analysis, the fully immune-augmented model (SOFA-2 + WBC + lymphocyte + NLR) significantly improved in discrimination compared with SOFA-2 alone (pooled AUC, 0.752 [95% CI, 0.716-0.787] vs. 0.741 [95% CI, 0.713-0.769]; p = 0.003). This improvement was paralleled by a strengthened association with mortality, evidenced by consistently higher standardized ORs for the augmented model across all cohorts. In terms of reclassification, the augmented model yielded a pooled total NRI of 0.051 (95% CI, 0.027-0.075; p < 0.001) (**Table 1**). Notably, the source of improvement varied by cohort: in MIMIC-IV and eICU, the net benefit was primarily driven by improved identification of nonsurvivors (NRI-events), whereas in MIMIC-III, the enhanced discrimination was driven by improved classification of survivors (NRI-non-events) (**Figure 1**). In a sensitivity analysis using 30-day mortality as the outcome, the immune-augmented model showed consistent improvements in discrimination and reclassification (**Supplementary Table S5**).

**Table 1:** Predictive performance of SOFA-1, SOFA-2, and immune-augmented SOFA-2 scores for in-hospital mortality across 3 cohorts.

Model	AUC (95% CI)	p-value^a^	OR (95% CI)^b^	NRI (95% CI)
MIMIC-IV (Test)				
SOFA-1	0.768 (0.756-0.781)	<0.001	2.590 (2.456-2.731)	0.076 (0.053- 0.098)
SOFA-2	0.747 (0.734-0.760)	Reference	2.344 (2.229-2.466)	Reference
SOFA-2+WBC+lymphocyte	0.755 (0.742-0.767)	<0.001	2.436 (2.313-2.565)	0.014 (-0.005-0.033)
SOFA-2+NLR	0.758 (0.746-0.771)	<0.001	2.450 (2.326-2.580)	0.020 (0.001-0.041)
SOFA-2+WBC+lymphocyte+NLR	0.760 (0.747-0.772)	<0.001	2.485 (2.358-2.618)	0.037 (0.018-0.057)
MIMIC-III				
SOFA-1	0.724 (0.709-0.740)	<0.001	2.337 (2.188-2.495)	0.115 (0.093-0.137)
SOFA-2	0.714 (0.698-0.730)	Reference	2.232 (2.093-2.381)	Reference
SOFA-2+WBC+lymphocyte	0.712 (0.697-0.728)	0.597	2.223 (2.084-2.371)	0.080 (0.059-0.098)
SOFA-2+NLR	0.720 (0.704-0.735)	0.048	2.261 (2.119-2.414)	0.063 (0.044-0.083)
SOFA-2+WBC+lymphocyte+NLR	0.717 (0.701-0.733)	0.299	2.266 (2.123-2.419)	0.076 (0.057-0.096)
eICU				
SOFA-1	0.757 (0.748-0.767)	0.002	2.609 (2.507-2.715)	-0.007 (-0.023-0.008)
SOFA-2	0.763 (0.754-0.772)	Reference	2.647 (2.545-2.753)	Reference
SOFA-2+WBC+lymphocyte	0.776 (0.767-0.785)	<0.001	2.782 (2.672-2.897)	0.034 (0.019-0.048)
SOFA-2+NLR	0.769 (0.761-0.778)	<0.001	2.714 (2.607-2.826)	0.017 (0.002-0.031)
SOFA-2+WBC+lymphocyte+NLR	0.779 (0.770-0.787)	<0.001	2.825 (2.712-2.944)	0.040 (0.026-0.053)
Total				
SOFA-1	0.750 (0.724-0.776)	0.268	Not available	0.061 (-0.010-0.131)
SOFA-2	0.741 (0.713-0.769)	Reference		Reference
SOFA-2+WBC+lymphocyte	0.748 (0.711-0.784)	0.112		0.042 (0.005-0.080)
SOFA-2+NLR	0.749 (0.720-0.779)	<0.001		0.033 (0.004-0.062)
SOFA-2+WBC+lymphocyte+NLR	0.752 (0.716-0.787)	0.003		0.051 (0.027-0.075)

AUC, area under the curve; SOFA, sequential organ failure assessment; WBC, white blood cell; NLR, neutrophil-to-lymphocyte ratio; OR, odds ratio; NRI, net reclassification improvement. WBC and lymphocyte refer to cell counts.

^a^ *P*-value for AUC comparison by DeLong’s test.

^b^ Odds ratio was reported per one standard deviation (SD) increment of the SOFA score.

## Discussion

While SOFA-2 is a robust tool for quantifying organ dysfunction, excluding the immune system for risk prediction warrants reconsideration. One goal of Sepsis-3 was to align the definition of sepsis with its pathogenesis which includes immune dysregulation as a primary driver of organ failure rather than a bystander (3). Omitting this domain leaves SOFA-2 disconnected from the host-response construct that the definition itself was built to capture. In practice, an immune-augmented score could refine risk stratification by identifying patients whose mortality risk is systematically under- or overestimated by SOFA-2 alone. Such a score is unlikely to function as a standalone diagnostic signal of infection, which remains a matter of integrated clinical judgment; its value would instead lie in informing the intensity of monitoring, the threshold for escalation of care, or eligibility for immunomodulatory trials.

The “lack of content validity” cited by the SOFA-2 task force for immune-based biomarkers likely stems from scoring methodology rather than the markers themselves. The provisional SOFA-2 immune criteria relied on reference ranges to define normality (1, 2). In infection, this approach penalizes adaptive leukocytosis, creating a ceiling effect where most patients accrue points. Our findings suggest that thresholds warrant redefinition using a data-driven approach—as we do for NLR—to identify “tipping points” that reflect maladaptive failure rather than physiological stress. At the extremes of the WBC distribution, however, the apparent plateau in mortality risk observed on the GAM curve more likely reflects sparse observations and correspondingly wider uncertainty than a genuine attenuation of risk. Data-driven threshold identification is therefore best interpreted alongside the underlying sample density, rather than from curve shape alone.

These findings are conceptual rather than prescriptive. The immune-augmented models were derived and validated in three retrospective cohorts; their thresholds and incremental performance require external validation before broader adoption. The principal contribution of this work is not a specific score, but the demonstration that immune function belongs in the pathophysiological construct organ dysfunction scores aim to capture. Future revisions of critical illness severity scores should incorporate an immune-function domain that is physiologically grounded and pragmatically accessible, built from multicenter cohorts spanning millions of patients and refined through structured expert consensus rather than derived *post hoc* from retrospective data.

Our study has limitations. First, the retrospective design and the use of US-centric databases may limit global generalizability. Second, because suspected infection in eICU was identified using an antibiotic-based proxy rather than the stricter Sepsis-3 operational criteria used in MIMIC-IV and MIMIC-III, some heterogeneity in case ascertainment across cohorts was unavoidable, which may limit the generalizability of the pooled meta-analysis to uniformly defined Sepsis-3 populations. Third, the high rate of missing NLR data (28%-50%) likely leads to an underestimation of the true prognostic power of immune augmentation. Fourth, because NLR is a non-specific inflammatory marker, the present findings are confined to critically ill patients with suspected infection and should not be extrapolated to non-infectious ICU populations without further validation. Finally, while we prioritized global feasibility, we acknowledge that specific biomarkers like HLA-DR cell surface expression may offer superior specificity in high-resource settings.

## Conclusions

In conclusion, the immune system remains a “missing link” in sepsis scoring systems. Here, we provide evidence that suggests future iterations should operationalize immune dysfunction through data-driven thresholds to ensure the score reflects the dysregulated host response.

## Data Availability

Publicly available datasets were analyzed in this study. This data can be found here: The datasets analyzed during the current study are available in the PhysioNet repository. Access to the data is restricted to credentialed users who have completed the required training and signed the data use agreement. The specific datasets can be accessed at: https://physionet.org/content/mimiciv/3.1/(MIMIC-IV), https://physionet.org/content/mimic3-carevue/1.4/(MIMIC-IIICareVue), and https://physionet.org/content/eicu-crd/2.0/(eICU-CRD).
