# Supplementary material for: Augmenting SOFA-2 with immune markers to capture dysregulated host response in patients with suspected infection
Source: Front Immunol. 2026 Jul 23;17:1904045. doi: 10.3389/fimmu.2026.1904045 (PMC13441755; doi:10.3389/fimmu.2026.1904045)
Supplement: Supplementary file 1 [file DataSheet1.pdf]

Supplementary figures and table

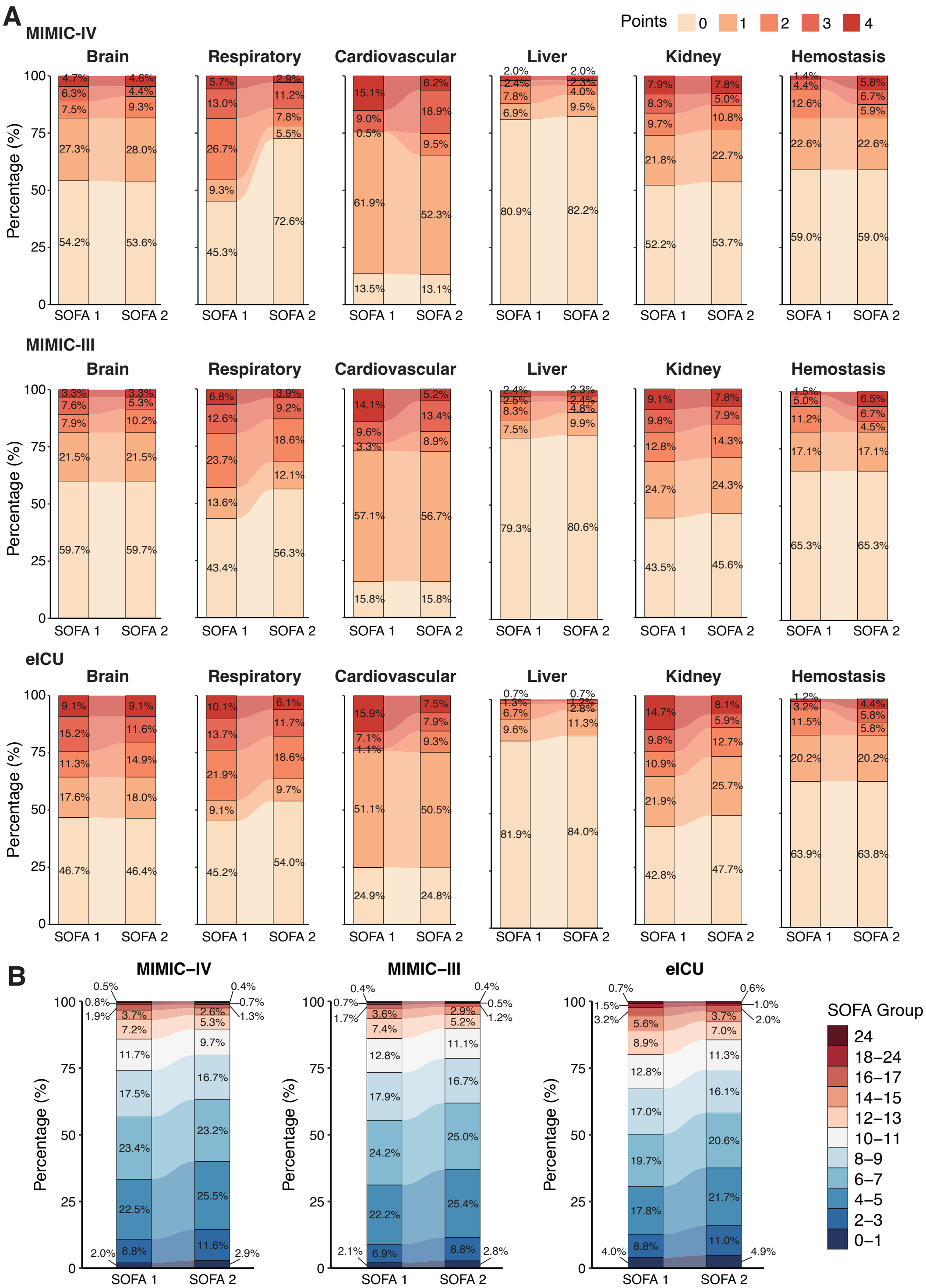

Figure S1. Distribution of SOFA-1 and SOFA-2 Scores Across 3 Cohorts. (A) Distribution of individual organ system subscores. **(B)** Distribution of the total SOFA scores grouped into risk ranges.

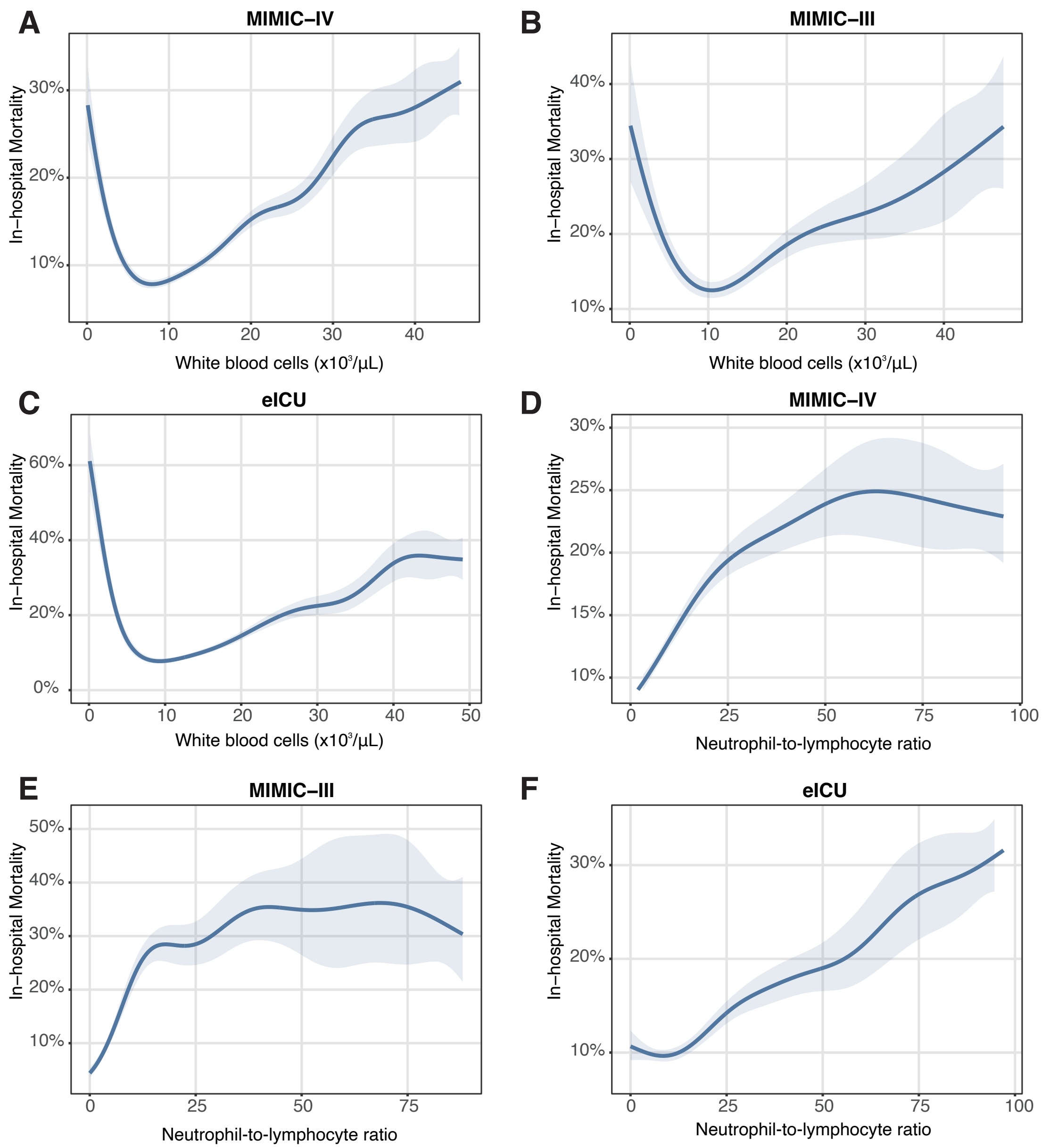

Figure S2. Association of White Blood Cell Count and Neutrophil-to-Lymphocyte Ratio With In-Hospital Mortality. A-C, Generalized additive model (GAM) plots showing the relationship between white blood cell count ($\times 10^3/\mu L$) and the probability of in-hospital mortality in the MIMIC-IV (**A**), MIMIC-III (**B**), and eICU (**C**) cohorts. **D-F,** GAM plots showing the relationship between neutrophil-to-lymphocyte ratio and the probability of in-hospital mortality in the MIMIC-IV (**D**), MIMIC-III (**E**), and eICU (**F**) cohorts. The solid blue lines represent the estimated smooth functions, and the shaded gray areas indicate the 95% CIs.

Table S1. List of antibiotics used to define suspected infection

Antibiotic class	Antibiotic
Aminoglycosides	Amikacin
Aminoglycosides	Gentamicin
Aminoglycosides	Streptomycin
Aminoglycosides	Tobramycin
Amphenicols	Chloramphenicol
Cephalosporins (1st generation)	Cefadroxil
Cephalosporins (1st generation)	Cefazolin
Cephalosporins (1st generation)	Cephalexin
Cephalosporins (2nd generation)	Cefuroxime
Cephalosporins (2nd generation, cephamycin)	Cefotetan
Cephalosporins (3rd generation)	Cefotaxime
Cephalosporins (3rd generation)	Cefpodoxime
Cephalosporins (3rd generation)	Ceftazidime
Cephalosporins (3rd generation)	Ceftriaxone
Cephalosporins (4th generation)	Cefepime
Fluoroquinolones	Ciprofloxacin
Fluoroquinolones	Levofloxacin
Fluoroquinolones	Moxifloxacin
Fluoroquinolones	Ofloxacin
Glycopeptides	Vancomycin
Lincosamides	Clindamycin
Macrolides	Azithromycin
Macrolides	Clarithromycin
Macrolides	Erythromycin
Monobactams	Aztreonam
Nitrofurans	Nitrofurantoin
Nitroimidazoles	Metronidazole
Penicillins (aminopenicillin + inhibitor)	Ampicillin-sulbactam
Penicillins (aminopenicillin)	Amoxicillin
Penicillins (aminopenicillin)	Ampicillin
Penicillins (antistaphylococcal)	Dicloxacillin
Penicillins (antistaphylococcal)	Nafcillin
Penicillins (antistaphylococcal)	Oxacillin
Penicillins (carboxypenicillin + inhibitor)	Ticarcillin-clavulanate
Penicillins (natural)	Penicillin G
Penicillins (ureidopenicillin + inhibitor)	Piperacillin-tazobactam
Rifamycins	Rifampin
Sulfonamides / folate-pathway inhibitors	Sulfadiazine
Sulfonamides / folate-pathway inhibitors	Trimethoprim

Sulfonamides / folate-pathway inhibitors	Trimethoprim-sulfamethoxazole
Tetracyclines	Doxycycline
Tetracyclines	Minocycline
Tetracyclines	Tetracycline

Table S2. Scoring criteria for immune markers integrated into the augmented SOFA-2 model

Scoring strategy	0	1	2	3	4
WBC+Lymphocyte count	WBC 4-10 **AND** (Lymphocytes unavailable or >0.5)	WBC 4-10 **AND** Lymphocytes ≤0.5 **OR** WBC 10-15	WBC 1-4 or 15-25 **AND** Lymphocytes unavailable	WBC 1-4 or 15-25 **AND** Lymphocytes ≤0.5	WBC <1 **OR** >25
NLR	NLR <7.8	NLR 7.8-14.5	NLR14.5-22.5	NLR 22.5-71.9	NLR ≥ 71.9
WBC+Lymphocyte count+NLR	WBC 4-10 **AND** (Lymphocytes unavailable or >0.5) **OR** NLR <7.8	WBC 4-10 **AND** Lymphocytes ≤0.5 **OR** WBC 10-15 **OR** NLR 7.8-14.5	WBC 1-4 or 15-25 **AND** Lymphocytes unavailable **OR** NLR14.5-22.5	WBC 1-4 or 15-25 **AND** Lymphocytes ≤0.5 **OR** NLR 22.5-71.9	WBC <1 **OR** >25 **OR** NLR ≥ 71.9

Abbreviations: WBC, white blood cell; NLR, neutrophil-to-lymphocyte ratio.

Uppercase **'AND'** and **'OR'** denote coordinate logical relationships between distinct conditions, whereas lowercase 'or' indicates alternatives within a single parameter. Each row represents one mutually exclusive augmentation strategy: each augmented SOFA-2 model incorporates exactly one of the three immune domains listed (0-4 points), resulting in a total possible score of 0-28 points.

Table S3. Missing data for WBC, absolute lymphocyte count, and NLR across cohorts

Cohort	WBC	Lymphocyte count	NLR
MIMIC-IV	0.76%	50.03%	50.12%
MIMIC-III	0.77%	37.41%	37.43%
eICU	0.97%	21.63%	28.2%

Abbreviations: WBC, white blood cell; NLR, neutrophil-to-lymphocyte ratio

Table S4. Baseline demographic and clinical characteristics of patients across 3 cohorts

Variable	eICU<br>N = 23,250	MIMIC-III<br>N = 6,143	MIMIC-IV<br>N = 43,446	p-value
Age	66.0 (55.0, 77.0)	68.7 (53.9, 80.0)	65.0 (54.0, 76.0)	<0.001[1]
Sex				<0.001[2]
Male	12,445 (54%)	3,337 (54%)	24,475 (56%)	
Female	10,805 (46%)	2,806 (46%)	18,971 (44%)	
BMI	27.6 (23.4, 33.3)	27.0 (23.3, 31.6)	27.5 (23.8, 32.3)	<0.001[1]
Admission type				<0.001[2]
Medical	17,568 (76%)	4,480 (73%)	29,803 (69%)	
Surgical	4,779 (21%)	1,614 (26%)	13,199 (30%)	
Others	903 (3.9%)	49 (0.8%)	444 (1.0%)	
SOFA-1	5.0 (3.0, 9.0)	5.0 (3.0, 8.0)	5.0 (3.0, 8.0)	<0.001[1]
SOFA-2	5.0 (2.0, 8.0)	4.0 (3.0, 7.0)	4.0 (2.0, 7.0)	<0.001[1]
GCS	14.0 (10.0, 15.0)	15.0 (13.0, 15.0)	15.0 (14.0, 15.0)	<0.001[1]
Lactate (mmol/L)	1.9 (1.2, 3.5)	2.3 (1.5, 4.0)	2.1 (1.4, 3.4)	<0.001[1]
WBC (×10^3/μL)	14.7 (10.7, 20.1)	13.4 (9.5, 18.7)	12.9 (9.0, 17.9)	<0.001[1]
Platelet ((×10^3/μL)	177.0 (124.0, 241.0)	189.0 (125.0, 264.0)	171.0 (116.0, 241.0)	<0.001[1]
Total bilirubin (mg/dL)	0.7 (0.4, 1.2)	0.7 (0.4, 1.7)	0.7 (0.4, 1.7)	<0.001[1]
PF	188.0 (110.0, 295.2)	266.7 (154.3, 409.5)	219.0 (128.0, 360.0)	<0.001[1]
Heart rate (bpm)	102.0 (89.0, 117.0)	106.0 (92.0, 120.0)	101.0 (89.0, 115.0)	<0.001[1]
Respiratory rate (bpm)	25.5 (21.5, 31.0)	27.0 (23.0, 31.5)	27.0 (23.0, 31.0)	<0.001[1]
mABP (mmHg)	62.0 (55.0, 69.5)	59.0 (53.0, 66.0)	60.0 (54.0, 66.0)	<0.001[1]
Temperature (°C)	37.7 (37.2, 38.3)	37.7 (37.1, 38.3)	37.3 (36.9, 37.8)	<0.001[1]

Variable	eICU<br>N = 23,250	MIMIC-III<br>N = 6,143	MIMIC-IV<br>N = 43,446	p-value
ICU LOS	2.1 (1.1, 4.2)	3.4 (1.9, 7.4)	2.3 (1.3, 4.7)	<0.001[1]
In-hospital mortality	3,050 (13%)	1,347 (22%)	5,245 (12%)	<0.001[2]
30-day mortality	Not available	1,433 (23%)	5,862 (13%)	<0.001[2]

Abbreviations: BMI, body mass index; SOFA, sequential organ failure assessment score; GCS, glasgow coma scale score; WBC, white blood cell; PF, PaO2/FiO2; mABP, mean arterial blood pressure; ICU, intensive care unit; LOS, length of stay.

Data are N (%) or median [interquartile range].

[1]Kruskal-Wallis rank sum test

[2]Pearson's Chi-squared test

Table S5. Predictive performance of SOFA-1, SOFA-2, and immune-augmented SOFA-2 scores for 30-day mortality across 2 cohorts

Model	AUC (95% CI)	p-value[a]	OR (95% CI)[b]	NRI (95% CI)
MIMIC-IV (Test)				
SOFA-1	0.752 (0.739-0.764)	<0.001	2.456 (2.335-2.583)	0.089 (0.067- 0.111)
SOFA-2	0.730 (0.717-0.743)	Reference	2.231 (2.125-2.341)	Reference
SOFA-2+WBC+lymphocyte	0.739 (0.726-0.752)	<0.001	2.320 (2.208-2.438)	0.002 (-0.015-0.018)
SOFA-2+NLR	0.743 (0.731-0.756)	<0.001	2.342 (2.228-2.461)	0.040 (0.022-0.058)
SOFA-2+WBC+lymphocyte+NLR	0.744 (0.732-0.757)	<0.001	2.367 (2.252-2.488)	0.048 (0.031-0.066)
MIMIC-III				
SOFA-1	0.720 (0.704-0.735)	0.001	2.290 (2.147-2.442)	0.071 (0.050-0.092)
SOFA-2	0.711 (0.695-0.726)	Reference	2.202 (2.066-2.346)	Reference
SOFA-2+WBC+lymphocyte	0.713 (0.696-0.728)	0.341	2.216 (2.075-2.354)	0.013 (-0.012-0.027)
SOFA-2+NLR	0.717 (0.702-0.732)	0.048	2.246 (2.107-2.396)	0.024 (0.004-0.045)
SOFA-2+WBC+lymphocyte+NLR	0.715 (0.700-0.731)	0.109	2.245 (2.105-2.393)	0.019 (-0.002-0.039)

Abbreviations: AUC, area under the curve; SOFA, sequential organ failure assessment; WBC, white blood cell; NLR, neutrophil-to-lymphocyte ratio; OR, odds ratio; NRI, net reclassification improvement. WBC and lymphocyte refer to cell counts.

[a] *P*-value for AUC comparison by DeLong's test.

[b] Odds ratio was reported per one standard deviation (SD) increment of the SOFA score.
